# 2,3-Bis(thio­phen-3-yl)quinoxaline

**DOI:** 10.1107/S1600536813004248

**Published:** 2013-02-16

**Authors:** Guy Crundwell, Jorge de Freitas

**Affiliations:** a Department of Chemistry, Central Connecticut State University, New Britain, CT 06053, USA

## Abstract

In the title compound, C_16_H_10_N_2_S_2_, the thienyl rings are inclined to one another by 62.71 (10)°, and are inclined by 63.94 (8) and 21.35 (8)° to the quinoline mean plane [maximum deviation = 0.031 (2) Å]. In the crystal, the mol­ecules pack in a herringbone pattern, with π–π stacking inter­actions [centroid–centroid distances = 3.7381 (15) and 3.7268 (15) Å].

## Related literature

For the synthesis of the title compound, and the crystal structure of the 2,3-di(thio­phen-2-yl)quinoxaline analogue, see: Crundwell *et al.* (2003 ▶). For the structure of a similar compound, see: Cantalupo *et al.* (2010 ▶).
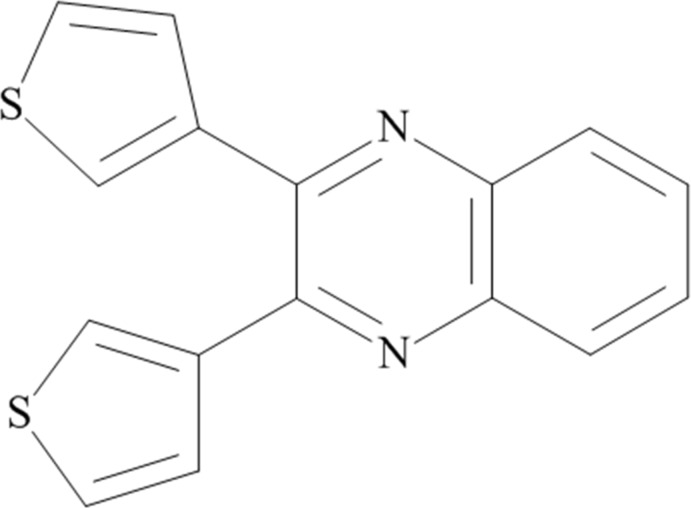


## Experimental

### 

#### Crystal data

C_16_H_10_N_2_S_2_
*M* *_r_* = 294.38
Monoclinic, 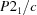

*a* = 15.966 (2) Å
*b* = 5.5741 (15) Å
*c* = 15.629 (4) Å
β = 98.25 (2)°
*V* = 1376.5 (6) Å^3^
*Z* = 4
Mo *K*α radiation
μ = 0.38 mm^−1^
*T* = 293 K
0.45 × 0.44 × 0.39 mm

#### Data collection

Oxford Diffraction Xcalibur Sapphire3 diffractometer
Absorption correction: multi-scan (*CrysAlis PRO*; Oxford Diffraction, 2009 ▶) *T*_min_ = 0.731, *T*_max_ = 1.000
30279 measured reflections
4745 independent reflections
2660 reflections with *I* > 2σ(*I*)
*R*_int_ = 0.087

#### Refinement

*R*[*F* ^2^ > 2σ(*F* ^2^)] = 0.057
*wR*(*F* ^2^) = 0.183
*S* = 0.92
4745 reflections
181 parameters
H-atom parameters constrained
Δρ_max_ = 0.44 e Å^−3^
Δρ_min_ = −0.50 e Å^−3^

### 

Data collection: *CrysAlis CCD* (Oxford Diffraction, 2009 ▶); cell refinement: *CrysAlis RED* (Oxford Diffraction, 2009 ▶); data reduction: *CrysAlis RED*; program(s) used to solve structure: *SHELXS97* (Sheldrick, 2008 ▶); program(s) used to refine structure: *SHELXL97* (Sheldrick, 2008 ▶); molecular graphics: *PLATON* (Spek, 2009 ▶); software used to prepare material for publication: *SHELXTL* (Sheldrick, 2008 ▶).

## Supplementary Material

Crystal structure: contains datablock(s) I, global. DOI: 10.1107/S1600536813004248/su2562sup1.cif

Structure factors: contains datablock(s) I. DOI: 10.1107/S1600536813004248/su2562Isup2.hkl

Supplementary material file. DOI: 10.1107/S1600536813004248/su2562Isup3.cml

Additional supplementary materials:  crystallographic information; 3D view; checkCIF report

## Figures and Tables

**Fig. 1.**
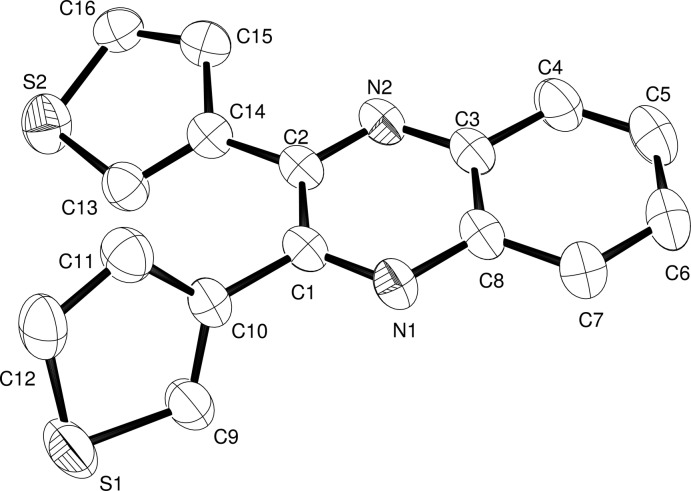
A view of the molecular structure of the title compound, with atom labelling. Displacement ellipsoids are drawn at the 50% probability level.

## supplementary crystallographic information

### Comment

In the title compound, Fig. 1, the quinoxaline moiety is flat, with a dihedral angle involving rings N1/N2/C1-C3/C8 and C3-C8 of 1.71 (9) °, and the two thienyl rings, S1/C9-C12 and S2/C13-C16, are inclined to the quinoxaline mean plane by 63.94 (8) and 21.35 (8) °, respectively. All bond lengths and angles fall within the typical ranges found in similar compounds (Cantalupo *et al.*, 2010; Crundwell *et al.*, 2003).

In the crystal, molecules pack in a herringbone pattern with π···π intermolecular contacts of 3.7381 (15) and 3.7268 (15) Å, for Cg1···Cg1^i^ and Cg2···Cg3^ii^, respectively [where Cg1 is ring S1/C9-C12; Cg2 is ring S2/C13-C16; Cg3 is ring N1/N2/C1-C3/C8; symmetry codes: (i) -x+1, -y+1, -z; (ii) x, y+1, z].

### Experimental

The title compound was prepared and purified according to literature methods (Crundwell *et al.*, 2003). Equal mole amounts of *o*-phenylenediamine (1.62 g, 15.0 mmol) and 3,3'-thenil (3.33 g, 15.0 mmol) were dissolved in 95% ethanol and heated in an Erlenmeyer flask in a hot water bath. Recrystallization of the crude product from boiling ethanol sufficiently purified the quinoxaline product as a pale white solid (3.71 g, 12.6 mmol; 84% yield; M.p. 403 K). Spectroscopic data for the title compound are available in the archived CIF.

### Refinement

Hydrogen atoms were included in calculated positions and included in the refinement in the riding motion approximation: C-H = 0.93 Å with *U*_iso_ = 1.2*U*_eq_(C).

### Crystal data

**Table:**

C_16_H_10_N_2_S_2_	*F*(000) = 608
*M**_r_* = 294.38	*D*_x_ = 1.420 Mg m^−^^3^
Monoclinic, *P*2_1_/*c*	Melting point: 406 K
Hall symbol: -P 2ybc	Mo *K*α radiation, λ = 0.71073 Å
*a* = 15.966 (2) Å	Cell parameters from 3293 reflections
*b* = 5.5741 (15) Å	θ = 4.1–33.0°
*c* = 15.629 (4) Å	µ = 0.38 mm^−^^1^
β = 98.25 (2)°	*T* = 293 K
*V* = 1376.5 (6) Å^3^	Block, white
*Z* = 4	0.45 × 0.44 × 0.39 mm

### Data collection

**Table:**

Oxford Diffraction Xcalibur Sapphire3 diffractometer	4745 independent reflections
Radiation source: fine-focus sealed tube	2660 reflections with *I* > 2σ(*I*)
Graphite monochromator	*R*_int_ = 0.087
Detector resolution: 16.1790 pixels mm^-1^	θ_max_ = 32.5°, θ_min_ = 4.2°
ω scans	*h* = −23→23
Absorption correction: multi-scan (*CrysAlis PRO*; Oxford Diffraction, 2009)	*k* = −8→8
*T*_min_ = 0.731, *T*_max_ = 1.000	*l* = −23→23
30279 measured reflections	

### Refinement

**Table:**

Refinement on *F*^2^	Primary atom site location: structure-invariant direct methods
Least-squares matrix: full	Secondary atom site location: difference Fourier map
*R*[*F*^2^ > 2σ(*F*^2^)] = 0.057	Hydrogen site location: inferred from neighbouring sites
*wR*(*F*^2^) = 0.183	H-atom parameters constrained
*S* = 0.92	*w* = 1/[σ^2^(*F*_o_^2^) + (0.1124*P*)^2^] where *P* = (*F*_o_^2^ + 2*F*_c_^2^)/3
4745 reflections	(Δ/σ)_max_ = 0.001
181 parameters	Δρ_max_ = 0.44 e Å^−^^3^
0 restraints	Δρ_min_ = −0.50 e Å^−^^3^

### Special details

**Table:**

Experimental. Spectroscopic data for the title compound: ^1^H NMR (300 MHz, (CD_3_)_2_CO) d 8.088 (m, 1H), 7.840 (m, 1H), 7.678 (dd, 1H),7.539 (dd, 1H), 7.331 (dd, 1H); ^13^C NMR (300 MHz,CDCl_3_) d 148.81, 140.86, 140.51, 129.88, 128.90, 128.60, 127.02, 125.50.
Geometry. All e.s.d.'s (except the e.s.d. in the dihedral angle between two l.s. planes) are estimated using the full covariance matrix. The cell e.s.d.'s are taken into account individually in the estimation of e.s.d.'s in distances, angles and torsion angles; correlations between e.s.d.'s in cell parameters are only used when they are defined by crystal symmetry. An approximate (isotropic) treatment of cell e.s.d.'s is used for estimating e.s.d.'s involving l.s. planes.
Refinement. Refinement of *F*^2^ against ALL reflections. The weighted *R*-factor *wR* and goodness of fit *S* are based on *F*^2^, conventional *R*-factors *R* are based on *F*, with *F* set to zero for negative *F*^2^. The threshold expression of *F*^2^ > σ(*F*^2^) is used only for calculating *R*-factors(gt) *etc*. and is not relevant to the choice of reflections for refinement. *R*-factors based on *F*^2^ are statistically about twice as large as those based on *F*, and *R*- factors based on ALL data will be even larger.

### Fractional atomic coordinates and isotropic or equivalent isotropic displacement parameters (Å^2^)

**Table:**

	*x*	*y*	*z*	*U*_iso_*/*U*_eq_	
N1	0.29116 (9)	0.2491 (3)	−0.02718 (9)	0.0397 (3)	
C1	0.29649 (11)	0.4316 (3)	0.02698 (11)	0.0360 (4)	
C2	0.22246 (11)	0.5650 (3)	0.04216 (11)	0.0373 (4)	
N2	0.14724 (10)	0.5169 (3)	−0.00197 (10)	0.0423 (4)	
C3	0.14154 (11)	0.3343 (4)	−0.06015 (11)	0.0406 (4)	
C4	0.06234 (12)	0.2759 (4)	−0.10817 (13)	0.0531 (5)	
H4	0.0146	0.3653	−0.1010	0.064*	
C5	0.05559 (14)	0.0887 (4)	−0.16501 (14)	0.0586 (6)	
H5	0.0034	0.0525	−0.1970	0.070*	
C6	0.12757 (15)	−0.0510 (4)	−0.17559 (14)	0.0543 (5)	
H6	0.1222	−0.1786	−0.2143	0.065*	
C7	0.20477 (13)	0.0005 (4)	−0.12934 (13)	0.0466 (5)	
H7	0.2516	−0.0929	−0.1361	0.056*	
C8	0.21308 (11)	0.1952 (3)	−0.07156 (11)	0.0391 (4)	
C9	0.43076 (12)	0.3380 (4)	0.12458 (12)	0.0478 (5)	
H9	0.4125	0.1877	0.1400	0.057*	
C10	0.38308 (11)	0.4901 (3)	0.07058 (11)	0.0367 (4)	
C11	0.42746 (12)	0.7043 (4)	0.05750 (12)	0.0469 (5)	
H11	0.4048	0.8291	0.0220	0.056*	
C12	0.50766 (12)	0.7072 (4)	0.10303 (14)	0.0535 (5)	
H12	0.5458	0.8328	0.1019	0.064*	
S1	0.52814 (3)	0.45345 (12)	0.16120 (4)	0.0617 (2)	
C13	0.28349 (12)	0.7946 (4)	0.17905 (12)	0.0453 (4)	
H13	0.3306	0.6973	0.1940	0.054*	
C14	0.22447 (11)	0.7587 (3)	0.10719 (11)	0.0387 (4)	
C15	0.15839 (13)	0.9348 (4)	0.10173 (13)	0.0459 (5)	
H15	0.1122	0.9381	0.0581	0.055*	
C16	0.17121 (12)	1.1020 (4)	0.16974 (13)	0.0439 (4)	
H16	0.1355	1.2304	0.1765	0.053*	
S2	0.26067 (4)	1.03661 (12)	0.23738 (4)	0.0598 (2)	

### Atomic displacement parameters (Å^2^)

**Table:**

	*U*^11^	*U*^22^	*U*^33^	*U*^12^	*U*^13^	*U*^23^
N1	0.0331 (7)	0.0495 (9)	0.0355 (7)	−0.0031 (6)	0.0019 (6)	−0.0029 (7)
C1	0.0306 (8)	0.0450 (10)	0.0321 (8)	−0.0010 (7)	0.0028 (6)	0.0029 (7)
C2	0.0324 (8)	0.0463 (11)	0.0324 (8)	−0.0016 (7)	0.0020 (6)	0.0040 (7)
N2	0.0338 (8)	0.0528 (10)	0.0387 (8)	−0.0007 (6)	0.0000 (6)	0.0033 (7)
C3	0.0352 (9)	0.0495 (11)	0.0353 (8)	−0.0035 (8)	−0.0007 (7)	0.0041 (8)
C4	0.0373 (10)	0.0663 (14)	0.0523 (11)	−0.0016 (9)	−0.0054 (8)	−0.0031 (10)
C5	0.0437 (11)	0.0759 (16)	0.0515 (12)	−0.0120 (10)	−0.0088 (9)	−0.0036 (11)
C6	0.0559 (12)	0.0628 (14)	0.0414 (10)	−0.0125 (10)	−0.0028 (9)	−0.0093 (10)
C7	0.0445 (10)	0.0530 (12)	0.0418 (10)	−0.0057 (8)	0.0043 (8)	−0.0053 (8)
C8	0.0346 (8)	0.0497 (11)	0.0322 (8)	−0.0066 (7)	0.0019 (6)	0.0027 (8)
C9	0.0388 (10)	0.0525 (12)	0.0490 (11)	−0.0023 (8)	−0.0045 (8)	0.0020 (9)
C10	0.0306 (8)	0.0470 (10)	0.0322 (8)	−0.0010 (7)	0.0030 (6)	−0.0044 (7)
C11	0.0424 (10)	0.0518 (12)	0.0461 (10)	−0.0055 (8)	0.0049 (8)	0.0037 (9)
C12	0.0373 (10)	0.0597 (13)	0.0635 (13)	−0.0132 (9)	0.0076 (9)	−0.0124 (11)
S1	0.0398 (3)	0.0779 (5)	0.0612 (4)	0.0005 (2)	−0.0134 (2)	−0.0057 (3)
C13	0.0437 (10)	0.0523 (11)	0.0398 (9)	0.0014 (9)	0.0052 (7)	−0.0036 (9)
C14	0.0358 (8)	0.0446 (10)	0.0368 (8)	−0.0011 (7)	0.0092 (7)	0.0008 (7)
C15	0.0408 (10)	0.0519 (12)	0.0462 (10)	0.0024 (8)	0.0104 (8)	0.0049 (9)
C16	0.0397 (9)	0.0428 (10)	0.0518 (11)	0.0044 (8)	0.0154 (8)	0.0022 (8)
S2	0.0572 (4)	0.0705 (4)	0.0532 (3)	−0.0039 (3)	0.0130 (3)	−0.0152 (3)

### Geometric parameters (Å, º)

**Table:**

N1—C1	1.318 (2)	C9—C10	1.351 (3)
N1—C8	1.370 (2)	C9—S1	1.7040 (19)
C1—C2	1.445 (2)	C9—H9	0.9300
C1—C10	1.487 (2)	C10—C11	1.418 (3)
C2—N2	1.324 (2)	C11—C12	1.373 (3)
C2—C14	1.480 (2)	C11—H11	0.9300
N2—C3	1.359 (2)	C12—S1	1.688 (2)
C3—C8	1.413 (3)	C12—H12	0.9300
C3—C4	1.413 (2)	C13—C14	1.373 (2)
C4—C5	1.365 (3)	C13—S2	1.697 (2)
C4—H4	0.9300	C13—H13	0.9300
C5—C6	1.417 (3)	C14—C15	1.435 (3)
C5—H5	0.9300	C15—C16	1.406 (3)
C6—C7	1.367 (3)	C15—H15	0.9300
C6—H6	0.9300	C16—S2	1.690 (2)
C7—C8	1.406 (3)	C16—H16	0.9300
C7—H7	0.9300		
			
C1—N1—C8	117.70 (15)	C10—C9—S1	112.20 (16)
N1—C1—C2	121.58 (16)	C10—C9—H9	123.9
N1—C1—C10	115.70 (15)	S1—C9—H9	123.9
C2—C1—C10	122.72 (16)	C9—C10—C11	111.71 (17)
N2—C2—C1	120.83 (17)	C9—C10—C1	123.50 (17)
N2—C2—C14	115.76 (16)	C11—C10—C1	124.71 (16)
C1—C2—C14	123.41 (15)	C12—C11—C10	112.53 (18)
C2—N2—C3	117.98 (16)	C12—C11—H11	123.7
N2—C3—C8	121.18 (16)	C10—C11—H11	123.7
N2—C3—C4	119.79 (17)	C11—C12—S1	111.33 (16)
C8—C3—C4	118.99 (18)	C11—C12—H12	124.3
C5—C4—C3	120.2 (2)	S1—C12—H12	124.3
C5—C4—H4	119.9	C12—S1—C9	92.21 (10)
C3—C4—H4	119.9	C14—C13—S2	112.35 (15)
C4—C5—C6	120.42 (19)	C14—C13—H13	123.8
C4—C5—H5	119.8	S2—C13—H13	123.8
C6—C5—H5	119.8	C13—C14—C15	111.18 (18)
C7—C6—C5	120.5 (2)	C13—C14—C2	127.79 (17)
C7—C6—H6	119.7	C15—C14—C2	121.00 (17)
C5—C6—H6	119.7	C16—C15—C14	112.50 (18)
C6—C7—C8	119.8 (2)	C16—C15—H15	123.8
C6—C7—H7	120.1	C14—C15—H15	123.8
C8—C7—H7	120.1	C15—C16—S2	110.37 (15)
N1—C8—C7	119.34 (17)	C15—C16—H16	124.8
N1—C8—C3	120.58 (17)	S2—C16—H16	124.8
C7—C8—C3	120.08 (16)	C16—S2—C13	93.60 (10)
